# An Advanced Electron Spin Resonance (ESR) Spin-Trapping and LC/(ESR)/MS Technique for the Study of Lipid Peroxidation

**DOI:** 10.3390/ijms131114648

**Published:** 2012-11-12

**Authors:** Yi Xu, Yan Gu, Steven Y. Qian

**Affiliations:** Department of Pharmaceutical Sciences, College of Pharmacy, Nursing and Allied Sciences, North Dakota State University, Fargo, ND 58108, USA; E-Mails: yi.xu@ndsu.edu (Y.X.); anna.gu@my.ndsu.edu (Y.G.)

**Keywords:** COX- and LOX-catalyzed lipid peroxidation, ESR spin-trapping, free radicals from cellular peroxidation, human colon cancer HCA7 colony 29 cells, LC/ESR and LC/MS combined technique

## Abstract

There are two types of nutritionally important polyunsaturated fatty acids (PUFAs), namely ω-6s and ω-3s. PUFAs and their metabolites generated from lipid peroxidation via cyclooxygenase (COX) and lipoxygenase (LOX) are believed to be involved in a variety of physiological and pathological processes in the human body. Both COX- and LOX-catalyzed PUFA peroxidation are complex events that generate a series of radicals, which may then bind proteins, target DNA/RNA, and lead to a number of biological changes. However, due to the lack of an appropriate method, it was not possible until recently to identify the short-lived PUFA-derived radicals in COX-/LOX-catalyzed peroxidation. Failure to characterize free radicals during peroxidation has greatly restricted our knowledge about COX/LOX biology in human health. Here we review the development and refinement of combined ESR spin trapping and LC/ESR/MS to characterize PUFA-derived radicals formed from *in vitro* (cell-free) peroxidation. We also present the most recent approach for studying peroxidation in cells which allows us to directly assess the potential bioactivity of PUFA-derived free radicals. This advanced technique has resulted in a major breakthrough in radical structural characterization, as well as assessment of free radical-associated cell growth response, thereby greatly improving our knowledge of PUFAs, COX-/LOX-catalyzed lipid peroxidation, and their related biological consequences.

## 1. Introduction

Polyunsaturated fatty acids (PUFAs) are one family of the most important components of cell membranes in living systems. Depending on the location of the first carbon–carbon double bond from the alkyl end of their backbones, two classes of PUFAs have been distinguished, namely ω-6s, e.g., dihomo-γ-linolenic acid (DGLA), linoleic acid (LA), and arachidonic acid (AA); and ω-3s, e.g., eicosapentaenoic acid (EPA), docospentaenoic acid (DPA), and docosahexenoic acid (DHA) [1–3]. Upon uptake from the diet or upon release from a cell membrane, the PUFAs will undergo enzyme-catalyzed lipid peroxidation, a well-known free radical-mediated metabolic process:

$$\begin{array}{ll} \text{LH}+{\text{X}}^{\cdot }\to {\text{L}}^{\cdot }+\text{XH} & \left(\text{Initiation}\right)\\ {\text{L}}^{\cdot }+{\text{O}}_{2}\to {\text{LOO}}^{\cdot } & \left(\text{Propagation}\right)\\ {\text{LOO}}^{\cdot }+\text{LH}\to \text{LOOH}+{\text{L}}^{\cdot } & \left(\text{Propagation}\right)\\ {\text{L}}^{\cdot }+{\text{L}}^{\cdot }\to \text{nonradical products} & \left(\text{Termination}\right)\\ {\text{L}}^{\cdot }+{\text{LOO}}^{\cdot }\to \text{nonradical products} & \left(\text{Termination}\right) \end{array}$$

The C-H bond at the *bis*-allylic methylene position of the PUFA is most vulnerable to oxidation due to its low dissociation energy (~75 kcal/mol) compared to a typical alkyl C-H bond (~101 kcal/mol) [4,5]. After H abstraction at this position by oxidants (X^•^) like hydroxyl radical and activated species in oxidizing enzymes, a lipid molecule (LH) will be converted into a carbon-centered lipid radical (L^•^). The L^•^ will then react with O_2_ to form peroxyl radical (LOO^•^), which can further attack other lipid molecules during the propagation process. In addition, the LOO^•^ can also be converted into other types of free radicals through Fenton-type reactions and β-scission [4,6–9].

Cyclooxygenase (COX) and lipoxygenase (LOX) are two families of lipid-peroxidizing enzymes which are responsible for the PUFA peroxidation pathway [10–20]. Their oxidase activities catalyze the conversion of PUFAs into various lipid derivatives (e.g., prostaglandins, thromboxanes), thus triggering a series of biochemical events which may lead to inflammatory disorders and various cancers [21–26].

The importance of PUFAs, as well as their metabolites, in maintaining hemostasis has attracted great research interest [27–36], and the investigation of the various radical intermediates generated during lipid peroxidation seems promising and may provide us with new insight into this area. However, there has been no appropriate methodology until the last few years for the detection and characterization of radical species due to their high reactivity and extremely short lifetimes. Failure to identify and quantify free radicals in biological systems has restricted our understanding of the biological implications of PUFAs in many diseases, especially in the inflammatory disorders and cancer.

In this paper, we review the development and refinement of a combined Electron Spin Resonance (ESR) spin-trapping and LC/ESR/MS technique for radical detection and characterization. We will focus mainly on its applications in a number of recent studies regarding COX/LOX-catalyzed lipid peroxidation. In addition, we will also present some recent data from an updated ESR spin trapping-LC/MS protocol, in which both the formation of free radicals from cellular lipid peroxidation and the radicals’ bioactivities under normal cellular growth conditions could be assessed at the same time and under the same experimental settings. All of these promising efforts towards the development, refinement and application of this combined technique will definitely improve our knowledge of PUFA-related bioactivities and COX/LOX-related cancer biology.

## 2. History of the ESR Spin-Trapping Technique

Electron Spin Resonance (ESR), also known as Electron Paramagnetic Resonance (EPR), is the conventional technique capable of directly detecting radical species. It has a principle similar to Nuclear Magnetic Resonance (NMR), but responds to the transition of electron spins between energy levels in unpaired electron-containing materials. This characteristic offers ESR a unique specificity for the measurement of free radicals. However, the high reactivity and short lifetimes of free radicals usually make them very unstable, particularly in biological systems, thus rendering them undetectable by ESR. To overcome this shortcoming, a spin-trapping technique came out in the early 1970s, in which the primary free radicals are allowed to react with spin-trapping agents (nitrone or nitroso compounds) to form a more stable radical spin-trapping adduct prior to direct ESR detection [37–40]. The application of the coupled spin-trapping ESR technique seems promising for the investigation of almost all types of radical species, as long as one can find a suitable spin trap agent for the particular radical of interest. As a consequence, many spin trap agents have been developed, including σ-phenyl-*tert*-butylnitrone (PBN), 2-methyl-2-nitrosopropane (MNP), σ-[4-pyridyl 1-oxide]-*N*-*tert*-butyl nitrone (POBN), 3,3,5,5-tetramethylpyrroline-*N*-oxide (TMPO), nitrosodurene and 5,5-dimethyl-1-pyrroline *N*-oxide (DMPO) [41–53].

Among all of the spin trap agents, due to its good spin-trapping ability and satisfactory solubility in biological media, POBN has been widely used to detect PUFA-derived free radicals formed from *in vitro* and *in vivo* lipid peroxidation (Reaction 1) [5,9,54–59]:


(Reaction 1)
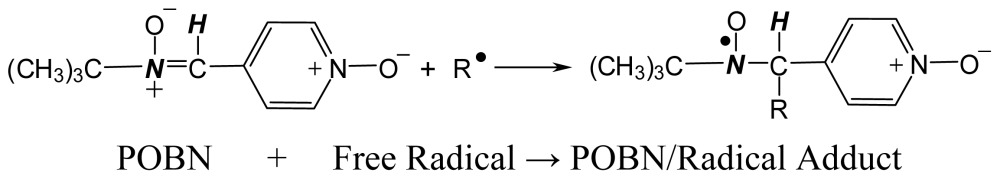


**Figure 1:**
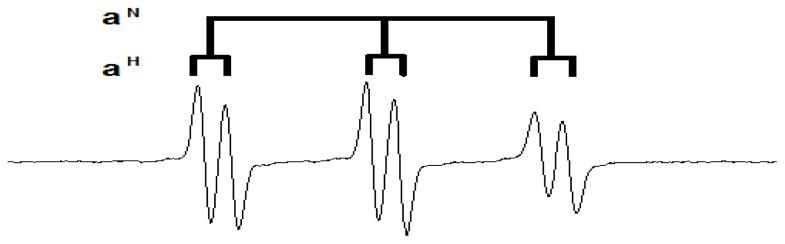
A typical ESR spectrum generated from a POBN radical adduct. The hyperfine coupling constants are σ^N^ ≈ 14.4~16.1 G and σ^H^ ≈ 2.2~2.8 G [9,60].

Although the appearance of the spin-trapping ESR technique provides us an easier way to work with radicals, the unambiguous structural assignment of radical species from ESR spectra still remains a problem. For instance, the POBN adducts of various radicals usually give six-line ESR spectrum with very similar hyperfine coupling constants: σ^N^ ≈ 14.4~16.1 G and σ^H^ ≈ 2.2~2.8 G (Figure 1) [9,60], because the splitting of the ESR signal mainly originates from the N and H atoms in the POBN molecule instead of from atoms in the different primary radical species. This feature makes it impossible to structurally distinguish each individual free radical trapped and generated from complex biological systems where more than one radical species are concurrently formed. Morever, unlike other comprehensive analytical techniques such as NMR and MS spectrometry, the ESR itself can hardly be viewed as a potent method for molecule identification because its unique parameters, such as hyperfine coupling constant and g value, provide structural information with insufficient specificity.

## 3. Development of the Combined ESR Spin-Trapping and LC/ESR/MS Technique

**Scheme 1:**
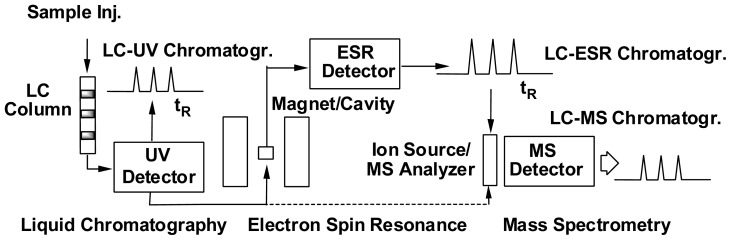
HPLC/ESR/MS combination for free radical detection and characterization. Spin-trapped free radicals with different structures are separated by an HPLC column according to their distinct chromatographic behavior, followed by ESR monitoring for radical confirmation and MS detection for structural identification.

In order to overcome the above limitations, a combination system employing ESR and HPLC as well as MS and/or tandem MS (MS^2^) has been developed to characterize individual radical species generated from PUFA peroxidation (Scheme 1). Briefly, spin trap adducts of radicals with different structures are first separated by a HPLC column according to their distinct chromatographic behavior, followed by ESR monitoring for radical confirmation and MS detection for structural identification. Note that in the online LC/ESR system, a time scan mode is used with the magnetic field fixed at the top of one line of the POBN adduct spectrum instead of running an entire magnetic field scan in offline ESR detection. With this practice, a series of absorption peaks matched to HPLC retention times can be observed, and each peak represents a structurally distinct free radical trapped by POBN.

The early effort in the development of this technique came from Iwahashi *et al.* in the late 1980’s [61,62]. The combined LC/ESR and LC/MS technique was employed in that work for the characterization of free radicals produced from PUFA peroxidation, epoxidation and β-scission. However, due to the unavailability of more advanced analytical techniques, some problems emerged in those early developments of the combined technique. For instance, the UV chromatogram of the system had never been reported due to poor chromatography resolution; the semipreparative LC column instead of the analytical one was employed, thus very large amounts of samples were required for these experiments, which is not feasible for biological systems; the ESR-active fractions were not detected as the corresponding *m*/*z* ions, but instead, the ions of the reduced forms of spin adducts were assigned based on their ESR-active peaks; some radical adducts, particularly those with a -COOH moiety (~p*K*a 5.0), could have very poor retention behavior due to the pH range (5–6) of the mobile phase (ammonium acetate) used for chromatographic separation; and a serious ESR-tuning problem appeared due to poor chromatograph resolution and low sensitivity arising from interface issues between the LC and ESR. This tuning problem often created artificial online ESR peaks during LC/ESR detection, thus raising the question of the method’s reliability.

Benefiting from great improvements in analytical technology, Qian *et al.* made a breakthrough in refining the LC/ESR and LC/MS methods to characterize PUFA-derived free radicals in many biological systems in the early 2000s [63–65]. The interface issues between online systems were tackled, and most problems encountered in previous developments have been successfully overcome: (1) replacing the classic ODS column (filled with a packing of octadecylsilyl groups chemically bonded to a silica gel) with a rapid resolution Eclipse column (extra dense bonding, high purity silica, and double endcapping) to achieve a better chromatographic resolution and sensitivity, allowing experiments to be conducted with analytic sampling and performance; (2) applying soft MS ionization and optimizing chromatographic separation to allow all ESR-active peaks to be detected as their corresponding *m*/*z* ions; (3) adding 1.0%–0.01% acetic acid (HOAc) to the mobile phase to maintain a weakly acidic pH to improve the retention behavior of all types of POBN adducts; and (4) adding a very small amount of tetrahydrofuran (THF) to the mobile phase to improve LC resolution. Besides, THF can also stabilize ESR tuning during LC/ESR measurements, perhaps attributable to its low dielectric constant. However, due to its corrosiveness, THF was not used as a mobile phase component in later combination LC/ESR and LC/MS systems [42,66–71].

**Scheme 2:**
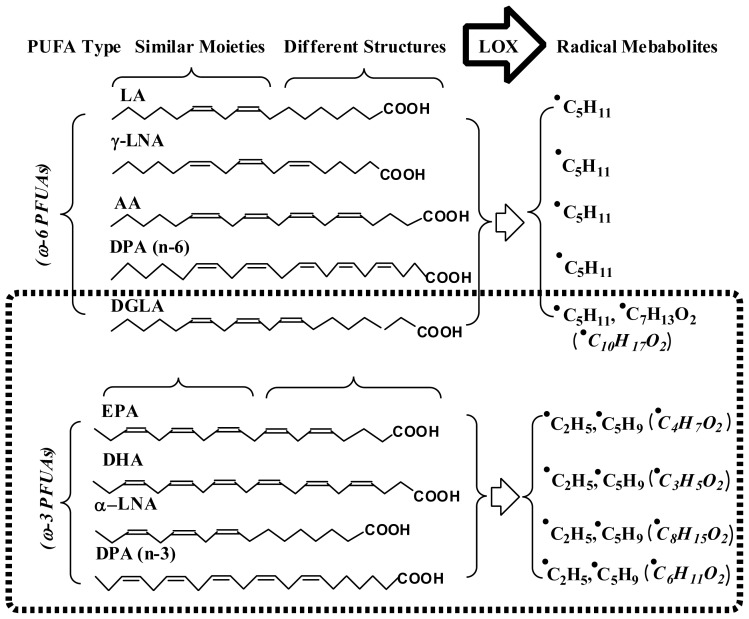
PUFA type-dependent free radical formation patterns of LOX-catalyzed lipid peroxidation. All of the listed free radical metabolites have been characterized in previous ESR spin-trapping and LC/ESR/MS studies [64–68]. Unlike the formation of one major radical product observed in LOX peroxidation of ω-6 PUFAs, LOX-catalyzed peroxidation of all ω-3 PUFAs along with DGLA (an exceptional ω-6) tended to produce multiple radical metabolites. Note that a similar metabolism pattern and the same pH-dependent oxygenation were observed in LOX-catalyzed DGLA (ω-6) peroxidation as in LOX-catalyzed ω-3 PUFA peroxidation.

The combination of spin trapping and LC/ESR/MS as refined via Qian *et al.* not only optimizes the method’s sensitivity and resolution, but also greatly improves the reliability of radical identification. Thus, it became a potent tool for later study on radical-mediated lipid peroxidation and its association with PUFA’s bioactivities. In fact, with the refined combined technique, almost all of the carbon-centered radical adducts formed from LOX catalyzed PUFA peroxidation (including both ω-6s and ω-3s) have been detected, structurally characterized and quantified (Scheme 2) [64–68]. These studies suggest that there is a PUFA type-dependent radical formation pattern in LOX-catalyzed lipid peroxidation: the peroxidation of ω-6 PUFAs (except DGLA) tend to produce one major radical product, while ω-3 PUFA peroxidation usually gives multiple radical metabolites. In addition, a change in the reaction pH was found to not only influence the total radical formation but also alter the preferred site of oxygenation in LOX-catalyzed PUFA peroxidation [68]. These PUFA type- and pH-dependent alterations of radical formation and oxygenation pattern may have significant implications and provide a basis for further investigations of fatty acids in cancer biology, particularly those peculiar to the acidic tumor environment (pH 6.5) compared to the normal physiological environment (pH 7.4).

## 4. Refined Combination of ESR Spin-Trapping and LC/MS Technique in Radical Characterization in *in vitro* (cell-free) COX-Catalyzed AA and DGLA Peroxidation

AA (20:4) and its upstream fatty acid DGLA (20:3) are two major 20-carbon ω-6 PUFAs in mammalian cells. During COX-catalyzed, free radical-mediated lipid peroxidation, DGLA and AA will be converted into 1-series Prostaglandins (PGs1) and 2-series Prostaglandin (PGs2), respectively, both of which are important signaling molecules implicated in a variety of physiological and pathological processes [11,16,18,21,22,24,26]. Interestingly, despite the structural similarity, AA and PGs2 are generally viewed as proinflammatory and protumorigenic factors, while DGLA and PGs1 may possess anticancer activity by virtue of suppressing oncogene expression and enhancing p53 activity [71–75]. Although this area has received a great deal of attention, the different radical-based mechanisms of DGLA peroxidation *vs.* AA peroxidation have never been clarified before due to the lack of appropriate methodology.

**Scheme 3:**
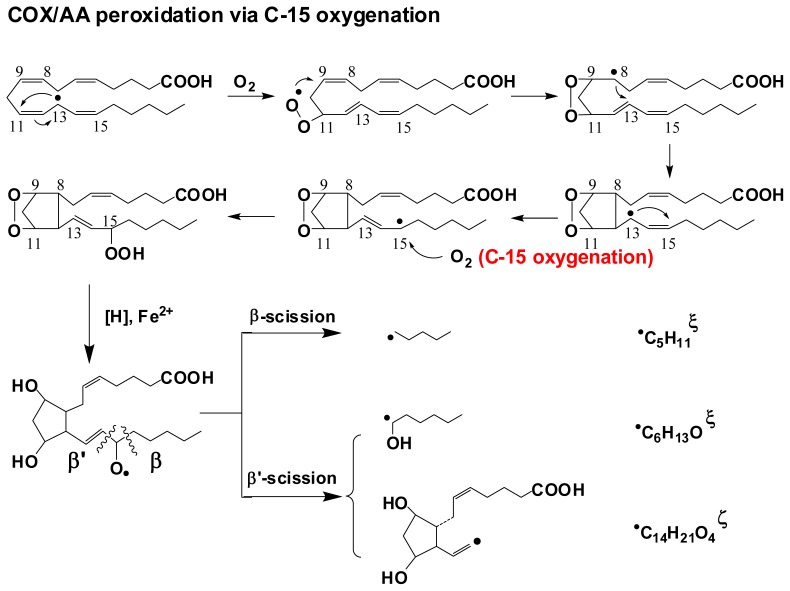
COX-catalyzed AA peroxidation pathway via C-15 oxygenation. Three major radical products (labeled with their corresponding molecular formulas) can be trapped by POBN, thus are screened by LC/MS as the radical adducts POBN/_•_C_14_H_21_O_4_, POBN/_•_C_6_H_13_O, and POBN/_•_C_5_H_11_, with *m*/*z* of 448, 296, and 266, respectively. ξ: Common radicals in COX/AA and COX/DGLA peroxidation. ξ: Exclusive radical in COX/AA peroxidation. All of the three major radical products can be observed in both the cell-free reactions and cellular experiments [71].

**Scheme 4:**
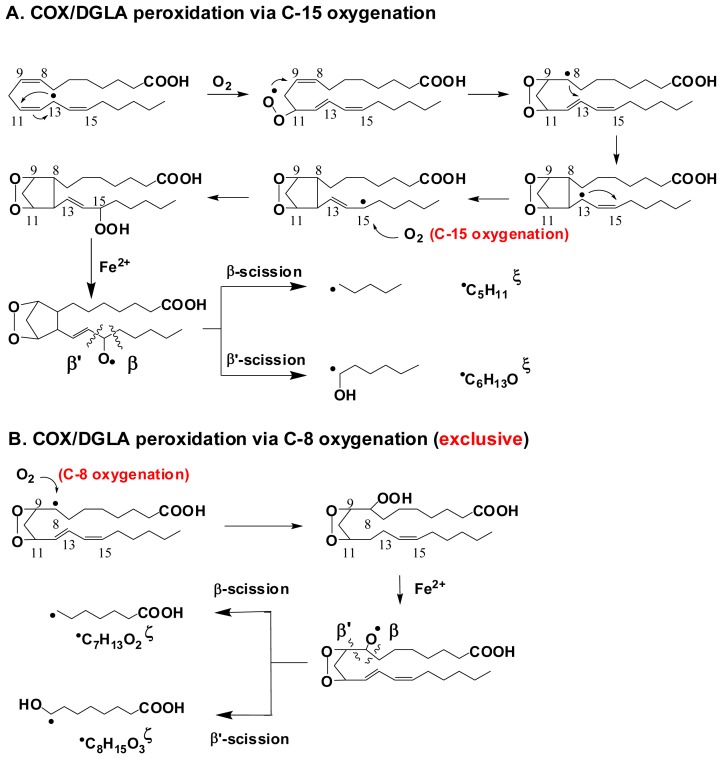
Proposed reaction pathways for COX-catalyzed DGLA peroxidation. (**A**) COX/DGLA peroxidation via C-15 oxygenation; (**B**) COX/DGLA peroxidation via C-8 oxygenation (the first two steps are the same as with C-15 oxygenation). The radical products (labeled with the corresponding molecular formulas) can be trapped by POBN, thus are screened by LC/MS as the radical adducts POBN/_•_C_6_H_13_O, POBN/_•_C_5_H_11_, POBN/_•_C_8_H_15_O_3_, and POBN/_•_C_7_H_13_O_2_ with m/z of 296, 266, 354, 324, respectively. ξ: Radicals common to COX/AA and COX/DGLA peroxidation. ξ: Radical exclusive to COX/DGLA peroxidation. All of the major radical products can be observed in both cell-free reactions and cellular experiments [71].

Using the combined ESR spin-trapping-LC/ESR and LC/MS technique, all the carbon centered free radicals formed in the COX/AA and COX/DGLA reaction systems were detected and structurally characterized in recent studies [69,70]. The results indicate that DGLA and AA share a common peroxidation mechanism and produce similar/identical radical products due to their structural similarity, while DGLA may also undergo a distinct reaction pathway to produce exclusive radical metabolites (Schemes 3,4).

In the COX/AA reaction system, an H atom is abstracted by COX, and then the peroxidase activity catalyzes the introduction of one molecule of O_2_ onto C-11 as the initiation step to form the C-9/C-11 endoperoxide, which is followed by C-8/C-12 cyclization. Then the second O_2_ molecule is introduced on C-15, a process called C-15 oxygenation, to produce 2-series PGs including PGG_2_, PGH_2_ and PGF_2_. During this process, a total of four types of radicals have been characterized using the combined LC/ESR/MS technique, including: ^•^C_20_H_34_O_5_ (not listed in Scheme 3) formed from the rearrangement of PGF_2_-type alkoxyl radicals; ^•^C_14_H_21_O_4_, a novel double-bonded carbon-centered radical derived from PGF_2_ via a special β′-scission; ^•^C_6_H_13_O, also derived from PGF_2_ via β′-scission; and ^•^C_5_H_11_, from β-scission of PGF_2_-type alkoxyl radicals (Scheme 3). After being spin trapped by POBN, all of these radical species were identified in LC/MS chromatography as ESR-active POBN/radical adducts: POBN/^•^C_20_H_34_O_5_ (*m*/*z* 548), POBN/^•^C_14_H_21_O_4_ (*m*/*z* 448), POBN/^•^C_6_H_13_O (*m*/*z* 296) and POBN/^•^C_5_H_11_ (*m*/*z* 266).

Similarly, C-15 oxygenation and β-scission as well as β′-scission (Scheme 4A), were also observed in the COX/DGLA system to produce the radicals ^•^C_5_H_11_ and ^•^C_6_H_13_O in common with COX/AA pathway. However, no analog of the double-bonded carbon-centered radical ^•^C_14_H_21_O_4_ was observed here, probably because the β′-scission took place during PGH_1_ formation instead of PGF_1_ formation [69]. Interestingly, in addition to the common reaction pathway, a distinct C-8 oxygenation pathway was proposed for the COX/DGLA reaction. This proposed pathway was confirmed by the identification of two radicals produced exclusively in the COX/DGLA system, observed as their corresponding adduct forms: POBN/^•^C_7_H_13_O_2_ (*m*/*z* 324) and POBN/^•^C_8_H_15_O_3_ (*m*/*z* 354) (Scheme 4B). The successful detection and characterization of these novel and exclusive free radicals from COX-catalyzed AA and DGLA peroxidation (cell-free) is very exciting.

## 5. Most Recent Update of ESR Spin-Trapping and LC/MS Protocol in Characterization of Free Radicals Formed from Cellular COX-Catalyzed AA and DGLA Peroxidation

**Figure 2:**
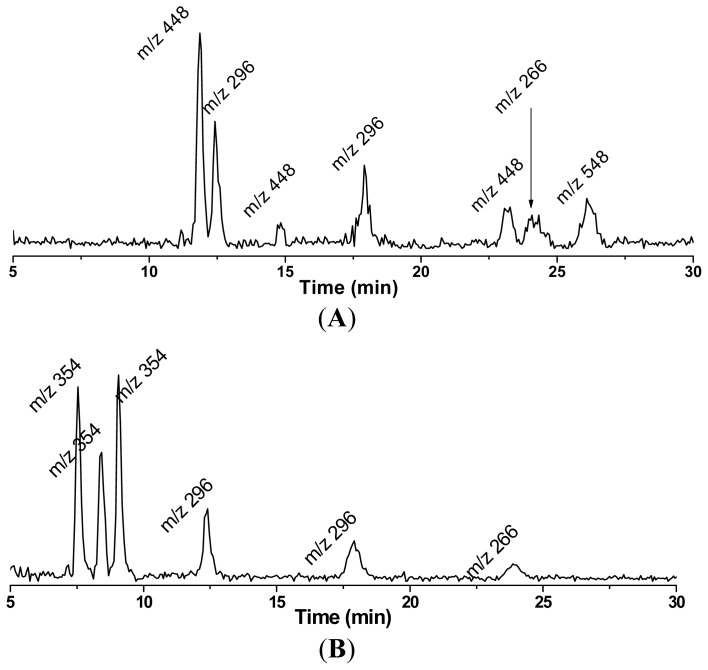
LC/MS chromatogram (EIC) of radical adducts from cellular PUFA peroxidation. (**A**) radical adducts from cells treated with AA; and (**B**) radical adducts from cells treated with DGLA. HCA-7 colony 29 cells were harvested and suspended in PBS. After being treated with high doses of POBN and PUFAs, the cells were incubated for 30 min. Then the reaction was stopped by mixing with ACN, and the supernatant was collected, centrifuged and condensed followed by LC/MS detection. In the COX/AA system, *m*/*z* 548, 448, 296, and 266 correspond to POBN/^•^C_20_H_34_O_5_, POBN/^•^C_14_H_21_O_4_, POBN/^•^C_6_H_13_O and POBN/^•^C_5_H_11_, respectively, which all come from C-15 oxygenation. In the COX/DGLA system, m/z 296 and 266 correspond to the common radical adducts POBN/^•^C_6_H_13_O and POBN/^•^C_5_H_11_ from C-15 oxygenation, while *m*/*z* 354 and 324 correspond to POBN/^•^C_8_H_15_O_3_ and POBN/^•^C_7_H_13_O_2_, two exclusive radical adducts from C-8 oxygenation in the COX/DGLA system.

To extend the application of the combined ESR spin trapping-LC/(ESR)/MS technique into complicated biological systems, Gu *et al.* further refined the method in a cell culture experiment [71]. With the use of human colon cancer HCA-7 colony 29 cells (with a high COX expression) in the PBS solution along with a high dose of POBN (50 mM) and PUFAs (500 μM) in a 30 min incubation [71], both the common and the differing pathways, as well as the formation of various radical products in AA and DGLA peroxidation, were confirmed for the first time (Figure 2). However, this protocol obviously suffers from two critical shortcomings: (1) only a short incubation time can be achieved due to the replacement of the cell medium by PBS; (2) due to the complexity of cellular conditions, a high dose of PUFAs as well as POBN must be used for cell treatment to get a satisfactory LC/MS signal intensity, and this excess of POBN and PUFA supplement can lead to cell death within one hour. As a result, it seems impossible to use this protocol to test cell proliferation, apoptosis and cell cycle distribution, thereby impeding assessment of the association between radical production and the biological activities of PUFAs.

**Scheme 5:**
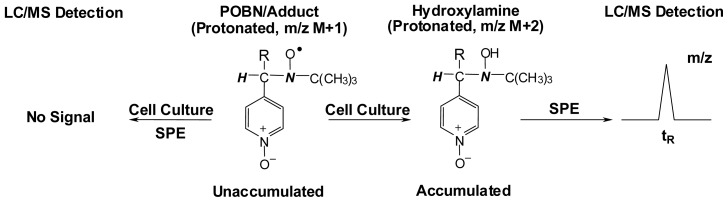
Refined ESR spin-trapping, SPE, LC/MS combined technique for characterization of radical adducts in reduced forms under normal cellular growth conditions. After a long incubation time, only the hydroxylamine of the radical adduct will accumulate, thus giving an LC/MS signal after SPE condensation.

In order to overcome the problems encountered in previous practices, the most updated combined technique was subjected to further refinement to characterize lipid-derived radicals under normal cellular growth conditions. It was proposed, due to the cellular reducing environment, that the POBN/radical adduct in ESR-active form could be easily reduced to hydroxylamine (Scheme 5) [71]. The hydroxylamine is a more stable redox form of radical adduct that can accumulate after a long incubation time and is readily detected by LC/MS.

Therefore, instead of directly detecting the ESR-active radical adducts, we subjected their reduced hydroxylamine forms to the LC/MS screen to profile the radical production in lipid peroxidation under normal cellular growth conditions. To achieve this, we employed a mixed-mode anion exchange SPE cartridge to extract and condense the hydroxylamines by taking advantage of the charge of the pyridyl-oxide group of the reduced POBN adduct. In this case, the cells were allowed to grow in cell culture media supplemented with much less POBN (20 mM) and PUFAs (100 μM). At different time points up to 48 h during a long incubation time, the cells together with media were collected and subjected to solid phase extraction (SPE) for condensation, followed by LC/MS and LC/MS_2_ detection.

**Figure 3:**
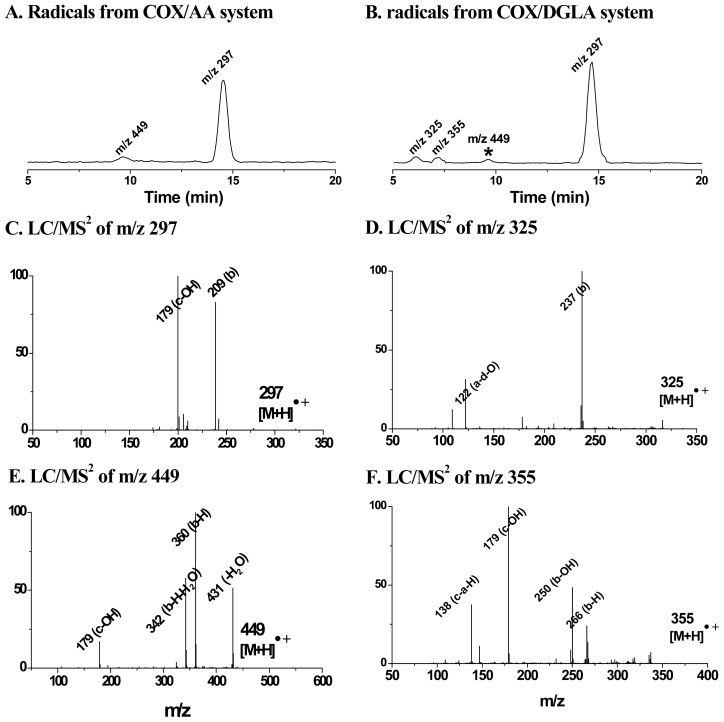
LC/MS and LC/MS^2^ detection of hydroxylamines from cellular PUFA peroxidation. (**A**) EIC of hydroxylamines in COX/AA cellular system, 8 h; (**B**) EIC of hydroxylamines in COX/DGLA cellular system, 12 h; (**C**) MS^2^ spectrum of hydroxylamines of *m*/*z* 297; (**D**) MS^2^ spectrum of hydroxylamines of *m*/*z* 325; (**E**) MS^2^ spectrum of hydroxylamines of *m*/*z* 449; and **F**) MS^2^ spectrum of hydroxylamines of *m*/*z* 355. Fragment a: M-[C(CH_3_)_3_]; b: M-[HO-N-C(CH_3_)_3_]; c: M-[R]; d: M-[POBN]. Note: in Figure 1B, the marked peak of *m*/*z* 449 in the COX/DGLA system comes from the conversion of DGLA to AA.

**Figure 4:**
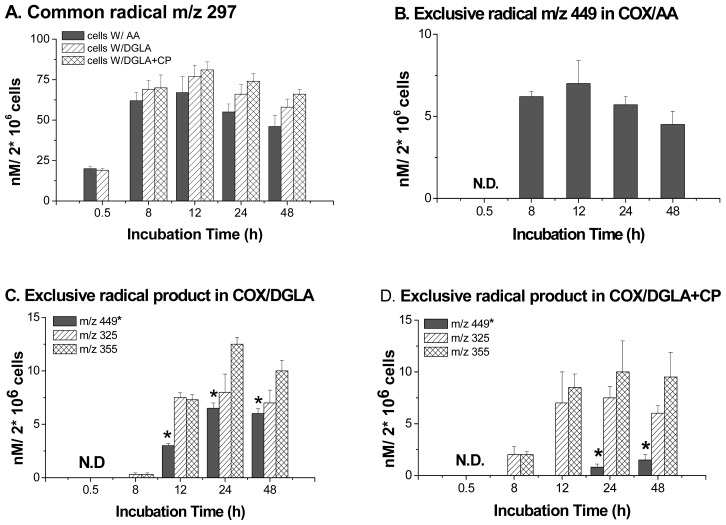
Profiles of reduced radical adducts in cellular PUFA peroxidation. (**A**) Profiles of the reduced common radical adduct *m*/*z* 297; (**B**) Profiles of the reduced exclusive radical adduct *m*/*z* 449 in cells treated with AA; (**C**) Profiles of reduced exclusive radical adducts in cells treated with DGLA; (**D**) Profiles of reduced exclusive radical adducts in cells treated by DGLA with CP-24879. Note: the asterisked *m*/*z* 449 in COX/DGLA and COX/DGLA+CP-24879 systems actually comes from COX/AA peroxidation because DGLA can be converted into AA by Δ-5 desaturase. When CP-24879, an inhibitor of Δ-5 desaturase, was introduced into the COX/DGLA system, the exclusive AA-derived hydroxylamine *m*/*z* 449 was barely detected. N.D.: Not detected.

As expected, the radical adducts in their reduced forms (hydroxylamines), but not the ESR-active forms, accumulated and were observed under these conditions. For instance, in the COX/AA system, the reduced forms of the POBN/^•^C_6_H_13_O and POBN/^•^C_14_H_21_O_4_ adducts were observed with the corresponding *m*/*z* values of 297 and 449, respectively, instead of their ESR-active forms (*m*/*z* 296 and *m*/*z* 448, respectively). Likewise, in the COX/DGLA system, the reduced forms of POBN/^•^C_6_H_13_O, POBN/^•^C_7_H_13_O_2_ and POBN/^•^C_8_H_15_O_3_ were observed up to 48 h with their corresponding *m*/*z* values of 297, 325 and 355, respectively (Figures 3,4). Dual spin-trapping experiments and MS^2^ detection were also conducted to further confirm the hydroxylamines as radical derivatives [71].

## 6. Direct Assessment of the Association between Radical Production and Cell Growth Response

With the most recently refined protocol, the radical products in COX/PUFA peroxidation were profiled under normal cell growth conditions, and their association with cell proliferation was assessed [71]. Here, the reduced form of the common radical adduct POBN/^•^C_6_H_13_O (*m*/*z* 297) was observed in both the COX/AA and COX/DGLA systems, and its production reached a peak at 12 h and started to decrease over the next two days. The hydroxylamine of the exclusive adduct in the COX/AA system, POBN/^•^C_14_H_21_O_4_ (*m*/*z* 449), was not observed until the 8 h-time point and reached a peak at 12 h. Similarly, the hydroxylamines of the two exclusive adducts in the COX/DGLA system, POBN/^•^C_7_H_13_O_2_ (*m*/*z* 325) and POBN/^•^C_8_H_15_O_3_ (*m*/*z* 355), also started to appear at 8 h and reached a peak at 24 h (Figure 4).

Interestingly, because the DGLA can be converted into AA by Δ-5 desaturase present in cells, the exclusive AA-derived hydroxylamine (POBN/^•^C_14_H_21_O_4_, *m*/*z* 449) was also observed in the COX/DGLA system, although no AA was introduced (Figures 3B,4). When CP-24879, an inhibitor of Δ-5 desaturase, was introduced into the COX/DGLA system, the exclusive AA-derived hydroxylamine was barely detected. The formation of the *m*/*z* 449 product seems a very likely response to cancer cell growth. Recent data has also shown that DGLA treatment was associated with sustained G_2_ phase cell cycle arrest compared to control after 8 h-incubation [71]. However, after one day of treatment, there was almost no difference in cell growth and cell cycle distribution between AA and DGLA treatments. Nevertheless, after one day of incubation, the addition of DGLA, together with CP-24879, resulted in sustained G_2_ phase cell cycle arrest and inhibited cell proliferation, e.g., a significant difference from AA or DGLA treatment alone (Table 1).

## 7. Summary

The refined combination of spin trapping with LC/(ESR)/MS not only optimizes the method’s sensitivity and resolution, but also greatly improves the reliability of radical identification. Thus, it becomes a potent tool for the study of radical-mediated lipid peroxidation, as well as its association with PUFA’s bioactivities. More important, the most recent ESR spin trapping and LC/MS technique with SPE has shown great advantages in the study of lipid peroxidation in cellular systems, particularly because it allows us to examine the radical production after a long incubation time and to assess its association with cancer cell growth. Thus, continued efforts to apply this advanced technique to exploring lipid peroxidation will definitely improve our knowledge of PUFAs’ bioactivities in cancer biology and various inflammatory disorders, as well as the radical-based mechanisms behind it.

## Figures and Tables

**Table 1:** Effect of PUFAs on cellular growth response.

Cells (HCA-7 Colony 29) Cultured with	Cell G_2_ Arrest		Cell Proliferation
	
	% in G_2_ Phase		% Viability
	
	8 h	24 h	48 h
Control a	29.7 ± 2.3	28.6 ± 1.6	100
AA(100 μM)	27.9 ± 1.9	30.5 ± 2.1	112.1 ± 3.9
DGLA(100 μM)	41.3 b ± 2.5	31.4 ± 2.0	109.1 ± 5.7
DGLA with 5.0 μM of CP	42.4 b ± 1.8	35.3 b ± 0.9	98.3 c ± 5.0

Cell cycle analysis (via PI staining) and cell proliferation assays (via MTS) were conducted using HCA-7 colony 29 cells treated with AA, DGLA and DGLA in combination with CP-24879 (Δ-5 desaturase inhibitor).

a : % cell viability was compared with the control group;

b : significantly different (*p* < 0.01) *vs.* control;

c : significantly different *vs.* treatment of AA and DGLA.

## Abbreviations

AA: arachidonic acid
COX: cyclooxygenase
DGLA: dihomo-γ-linolenic acid
DHA: docosahexaenoic acid
DMPO: 5,5-dimethyl-1-pyrroline *N*-oxide
DPA: docosapentaenoic acid
EIC: extracted ion current
EPA: eicosapentaenoic acid
ESR: electron spin resonance
HOAc: acetic acid
HPLC (LC): high performance liquid chromatography
LA: linoleic acid
LNA: linolenic acid
LOX: lipoxygenase
MS: mass spectrometry
MNP: 2-methyl-2-nitrosopropane
NMR: nuclear magnetic resonance
PBN: σ-phenyl-*tert*-butylnitrone
POBN: σ-[4-pyridyl-1-oxide]-*N-tert*-butylnitrone
PG: prostaglandin
PUFAs: polyunsaturated fatty acids
THF: tetrahydrofuran
TMPO: 3,3,5,5-tetramethylpyrroline-*N*-oxide
*t*_R_: retention time
